# CD45RB Is a Novel Molecular Therapeutic Target to Inhibit Aβ Peptide-Induced Microglial MAPK Activation

**DOI:** 10.1371/journal.pone.0002135

**Published:** 2008-05-14

**Authors:** Yuyan Zhu, Huayan Hou, William V. Nikolic, Jared Ehrhart, Elona Rrapo, Paula Bickford, Brian Giunta, Jun Tan

**Affiliations:** 1 Rashid Laboratory Developmental Neurobiology, Silver Child Development Center, Department of Psychiatry and Behavioral Medicine, University of South Florida College of Medicine, Tampa, Florida, United States of America; 2 Center for Excellence in Aging and Brain Repair, Department of Neurosurgery, University of South Florida College of Medicine, Tampa, Florida, United States of America; 3 Veterans Administration Hospital, Research Service, University of South Florida College of Medicine, Tampa, Florida, United States of America; University of North Dakota, United States of America

## Abstract

**Background:**

Microglial activation, characterized by p38 MAPK or p44/42 MAPK pathway signal transduction, occurs in Alzheimer's disease (AD). Our previous studies demonstrated CD45, a membrane-bound protein tyrosine phosphatase (PTP), opposed β-amyloid (Aβ) peptide-induced microglial activation *via* inhibition of p44/42 MAPK. Additionally we have shown agonism of the RB isoform of CD45 (CD45RB) abrogates lipopolysaccharide (LPS)-induced microglial activation.

**Methodology and Results:**

In this study, CD45RB modulation of Aβ peptide or LPS-activated primary cultured microglial cells was further investigated. Microglial cells were co-treated with “aged” FITC-Aβ_1–42_ and multiple CD45 isoform agonist antibodies. Data revealed cross-linking of CD45, particularly the CD45RB isoform, enhances microglial phagocytosis of Aβ_1–42_ peptide and inhibits LPS-induced activation of p44/42 and p38 pathways. Co-treatment of microglial cells with agonist CD45 antibodies results in significant inhibition of LPS-induced microglial TNF-α and IL-6 release through p44/42 and/or p38 pathways. Moreover, inhibition of either of these pathways augmented CD45RB cross-linking induced microglial phagocytosis of Aβ_1–42_ peptide. To investigate the mechanism(s) involved, microglial cells were co-treated with a PTP inhibitor (potassium bisperoxo [1,10-phenanthroline oxovanadate; Phen]) and Aβ_1–42_ peptides. Data showed synergistic induction of microglial activation as evidenced by TNF-α and IL-6 release; both of which are demonstrated to be dependent on increased p44/42 and/or p38 activation. Finally, it was observed that cross-linking of CD45RB in the presence of Aβ_1–42_ peptide, inhibits co-localization of microglial MHC class II and Aβ peptide; suggesting CD45 activation inhibits the antigen presenting phenotype of microglial cells.

**Conclusion:**

In summary, p38 MAPK is another novel signaling pathway, besides p44/42, in which CD45RB cross-linking negatively regulates microglial Aβ phagocytosis while increasing potentially neurotoxic inflammation. Therefore, agonism of CD45RB PTP activity may be an effective therapeutic target for novel agents to treat AD due to its Aβ lowering, and inflammation reducing, properties that are particularly targeted at microglial cells. Such treatments may be more effective with less potential to produce systemic side-effects than therapeutics which induce non-specific, systemic down-regulation of inflammation.

## Introduction

Classic findings of AD on autopsy are senile plaques, neurofibrillary tangles, cerebral amyloid angiopathy, neuronal loss, neuronal cytoskeleton disruption with altered connectivity, and widespread synaptic loss. Although the precise etiology of AD remains uncertain, it may result from an elevation in brain β-amyloid (Aβ) protein[1]. Indeed, Aβ peptide generation and aggregation as plaques are key pathological events in the development of AD [2], [3]. They have been extensively studied and evidenced to be neurotoxic, as they are reported mediators of inflammation [4], [5].

Activated microglia also play a critical role in the inflammatory processes of AD, as they secrete cytokines in response to Aβ, including tumor necrosis factor α (TNF-α) and interleukin-1 β (IL-1β) which promote neurodegeneration [6], [7]. However, current anti-inflammatory therapeutics directed against AD, including nonsteroidal anti-inflammatory drugs (NSAIDs), only partially suppress microglial activation [8], [9]. Furthermore to date, randomized, double-blind clinical trials of NSAIDS in AD patients have been negative [10], one trial on secondary prevention has not been promising, and there have been no prevention trials completed. Thus, a more viable therapeutic strategy may be combination of NSAIDs with specific inhibitors of microglial activation [11].

One viable target on microglia is the CD40-CD40L signaling pathway. This pathway is involved in both T-cell and microglial cell activation [12]–[15]. We demonstrated ligation of microglial CD40 synergistically enhanced autocrine activation by Aβ peptide [13]. As such, this pathway can be effectively used as a target for opposing both T-cell [15] and microglial activation.

To explore the possibility of immunomodulating CD40 activity, we showed that CD45, a protein tyrosine phosphatase (PTP), activation inhibits CD40L-induced microglial activation *via* down-regulation of the p44/42 mitogen activated protein kinase (MAPK) pathway [14]. Indeed, a synergistic induction of microglial TNF-α and nitric oxide (NO) release was found to be dependent on activation of p44/42 MAPK. Further, co-treatment with a PTP inhibitor [potassium bisperoxo (1,10-phenanthroline oxovanadate; phen)] and Aβ peptides resulted in microglia-induced neuronal injury. Conversely, stimulation of microglial CD45 by CD45 antibody markedly inhibited these effects *via* inhibition of p44/42 MAPK, suggesting CD45 is a negative regulator of microglial activation. Accordingly, primary cultured microglia from CD45-deficient mice displayed hyper-responsiveness to Aβ, as evidenced by TNF-α release, NO production, and neuronal injury. *In vivo*, brains from a transgenic mouse model of AD [Swedish APP-overexpressing (Tg2576) mice] deficient for CD45 further confirmed increased production of TNF-α compared with Tg2576 mice [11].

CD45 is a haemopoietic cell specific PTP, essential for antigen receptor-mediated signaling in T and B cells [16], as well as microglia [17], [18]. It modulates signaling through cytokine receptors as well as cellular adhesion [19]–[21]. The CD45 protein is encoded by a single gene (*PTPRC*; protein tyrosine phosphatase, receptor-type C) and different isoforms can be cleaved by alternative splicing of three variable exons: A, B, and C [22]. Expression of the various isoforms is tightly regulated by cell type and state of activation. In humans, naive/unprimed T cells express high molecular weight isoforms containing exon A, however upon activation CD45RB is the predominant form[11], [23]. CD45 may be particularly salient to the clinical features of AD since microglia express it in the frontal cortex and hippocampus of normal aging individuals. This expression level is markedly elevated in these brain regions in AD cases [24]. Furthermore, in an animal model of neurodegeneration, upregulation of PTP signaling in activated microglia was found in and around degenerating brain regions [25]. As phosphorylation of the related cytoplasmic serine threonine kinase, p38 MAPK, is also a response to Aβ and inflammatory molecules, it is likely that p38 MAPK activation is also critical to the disease cascade. Indeed activation of the p38 pathway in microglia or neurons may stimulate the production of inflammatory mediators, thereby contributing to the degeneration or further activation of these cells. Together these data led us to investigate the possible involvement of CD45RB PTP signaling as a putative down-regulator of microglial p38 activation in response to Aβ peptides.

## Results

### The cross-linking of CD45RB enhances microglial phagocytosis of Aβ_1–42_ peptide

**Figure 1:**
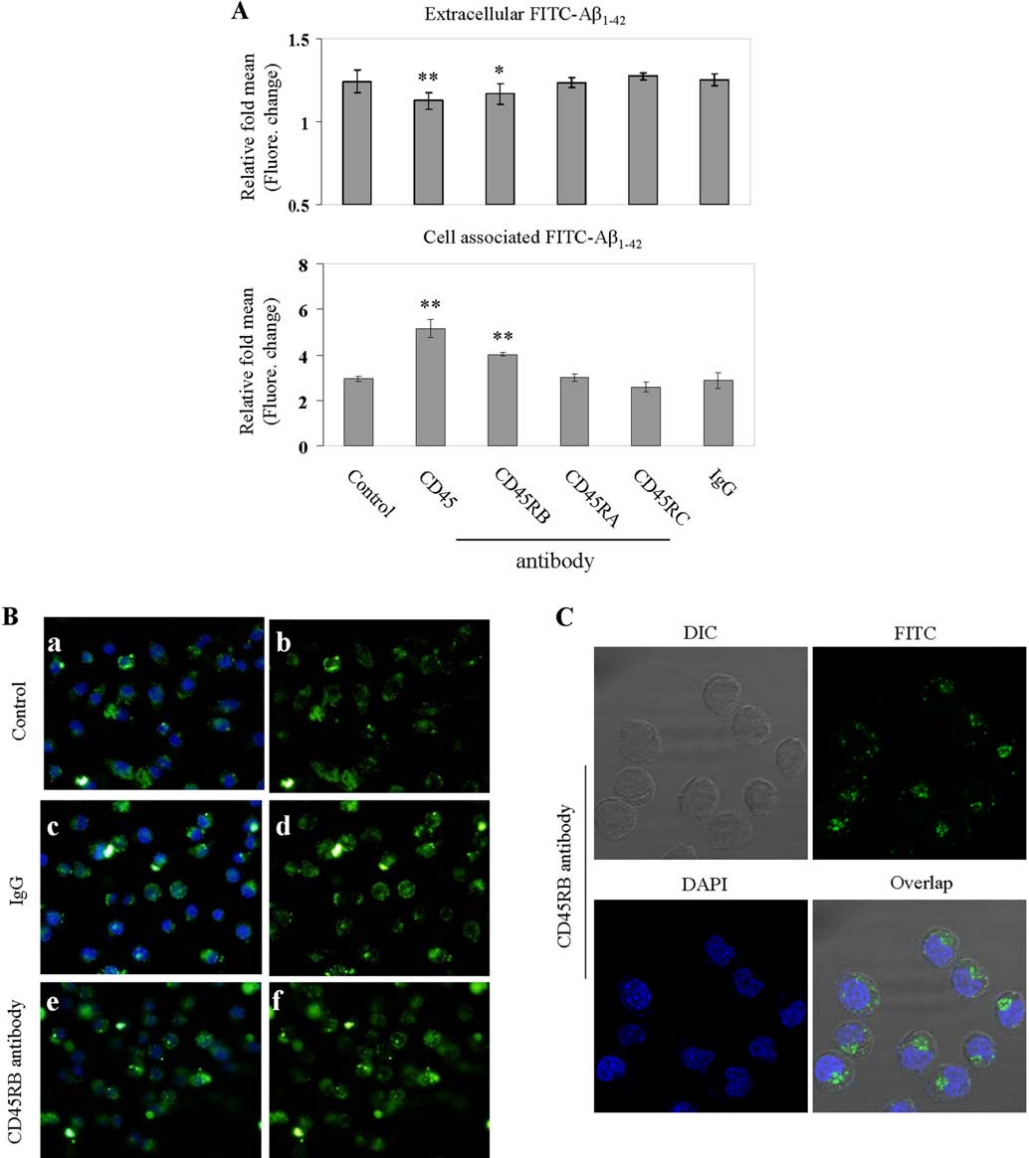
The cross-linking of CD45RB enhances microglia phagocytosis of Aβ_1–42_ peptide. (A) Cell supernatants and lysates were analyzed for extracellular (top panel) and cell-associated (bottom panel) FITC-Aβ_1–42_ using a fluorometer. Data are represented as the relative fold of mean fluorescence change (mean±SD), calculated as the mean fluorescence for each sample at 37°C divided by mean fluorescence at 4°C (n = 6 for each condition presented). One-way ANOVA followed by *post-hoc* comparison showed a significant between-group difference (*P<0.05, **P<0.001 compared with control or isotype-control IgG). (B) Subsequently, fluorescence microscope examination was performed using a 40× objective with appropriate filter selection. The dark field images (panels a, c and e) show the fluorescence of FITC labeled Aβ_1–42_ and DAPI-labeled nuclear stain, whereas, *b, d* and *f* show only the FITC Aβ_1–42_ stain of the same fields. (C) In parallel experiments, microglial cells were treated with 1 µM “aged” FITC-Aβ_1–42_ and CD45RB antibody for 2 h. Following treatment, these cells were fixed and stained with DAPI. The images were analyzed by confocal microscope and show FITC-Aβ_1–42_ (green staining) localized within the cytoplasm of microglia cells.

Microglial phagocytosis of Aβ is most likely a terminal event leading to removal of β-amyloid from the brain parenchyma [26]. In our recent studies we identified that CD45 cross-linking opposes both Aβ and CD40L-stimulated microglial activation [14], [27]. Thus to examine the functionality of CD45 isoforms, we first evaluated whether cross-linking with CD45 specific agonist antibodies (CD45RA, CD45RB and CD45RC antibodies) could modulate microglial uptake of Aβ. “Aged” FITC-tagged Aβ_1–42_ (500 nM) was added to primary cultured microglial cells for 2 h in the absence (control), presence of either variant CD45 specific antibodies [CD45 (total), CD45RA, CD45RB, CD45RC], or IgG isotype-control (2.5 µg/mL). As a control for non-phagocytic incorporation of Aβ by microglia, microglial cells were incubated at 4°C in parallel cell culture plates under the same treatment conditions described above. Cell supernatants and lysates were analyzed for extracellular and cell-associated FITC-Aβ using a fluorometer. As shown in Fig. 1A (top and bottom panel), the cross-linking of CD45, mainly isoform CD45RB, enhances microglia phagocytosis of Aβ_1–42_ peptide. This enhancement of Aβ_1–42_ phagocytosis peptides was verified by quantitative immunofluorescence assay (Fig. 1B). In a parallel experiment, results further showed microglial phagocytosis of Aβ_1–42_ peptide was localized within the cytoplasm of microglial cells (Fig. 1C).

### LPS-mediated microglial p38 and p44/42 MAPK activation hinders microglial phagocytosis of Aβ_1–42_ peptide

**Figure 2:**
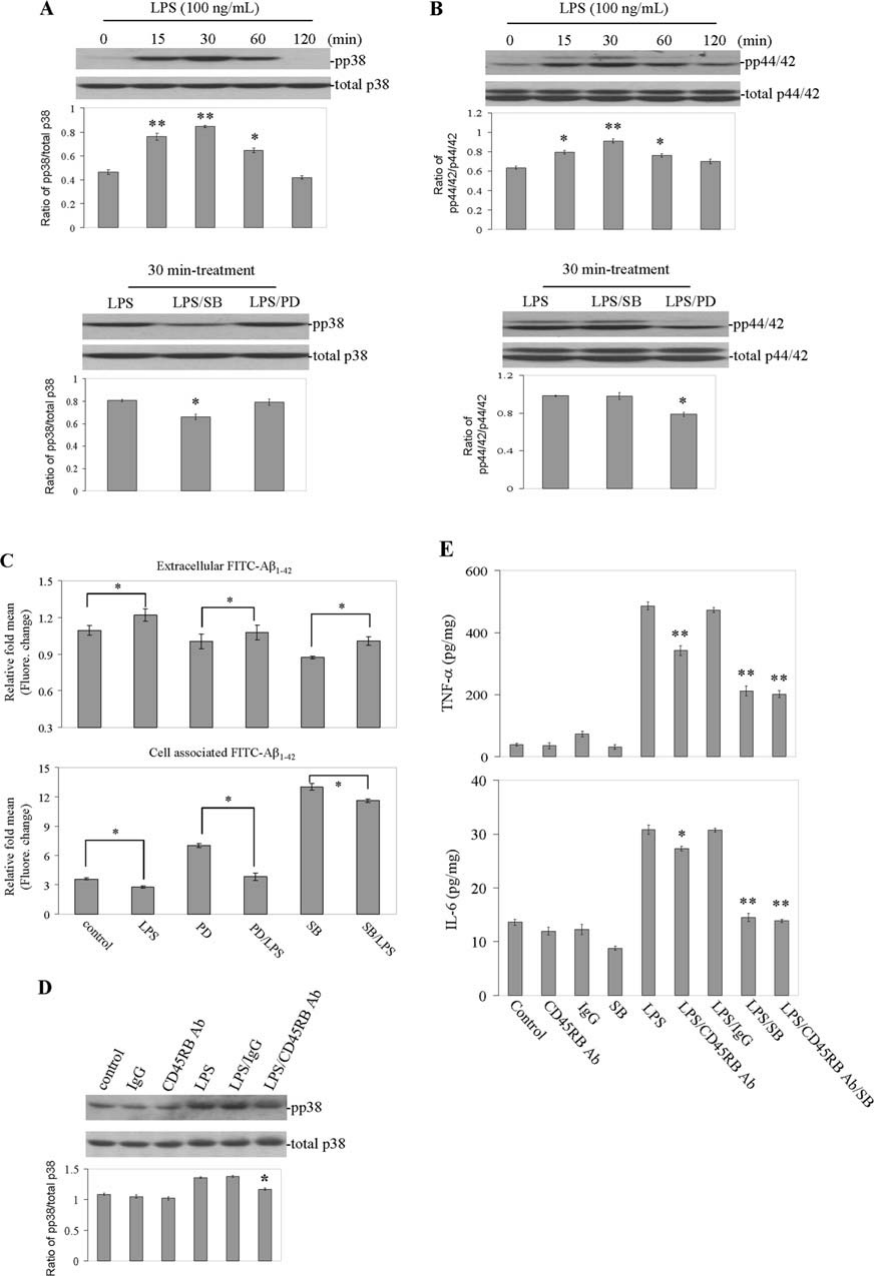
Activated p38 MAPK and/or p44/42 MAPK by LPS negatively affects microglial phagocytosis of Aβ_1–42_ peptide. Microglial treatment conditions are indicated and are further described in Materials and Method. Cell lysates were analyzed by Western immunoblotting using specific antibodies that recognize phosphorylated or total p38 MAPK and/or p44/42 MAPK at the indicated time points (A and B, top panel). Phosphorylation of both p38 MAPK and/or p44/42 MAPK after treatment with LPS was inhibited by SB203580 or PD98059 (A and B, bottom panel). Histograms below the immunoblots represent the mean band density ratio±1 SD (pp38 MAPK/total p38 MAPK and/or pp44/42 MAPK/total p44/42 MAPK; n = 3 for each condition presented; *P<0.05, **P<0.001 compared with control). (C) Microglial phagocytosis of Aβ_1–42_ peptide after pre-treatment with PD98059 or SB203580 for 1 h, then co-treated with “aged” FITC-tagged Aβ_1–42_ and LPS. Supernatants and cell lysates were analyzed for extracellular (top panel) and cell-associated (bottom panel) FITC-Aβ_1–42_ using a fluorometer (*P<0.05, **P<0.001). (D) Phosphorylation of p38 MAPK and inhibition of this effect by CD45RB antibody. Histograms below the immunoblots represent the mean band density ratio±1 SD (pp38/total p38; n = 3 for each condition presented; *P<0.05 compared with LPS or LPS/IgG). (E) Microglial activation is evidenced by mean TNF-α and IL-6 release±1 SD (n = 3 for each condition presented; *P<0.05; **P<0.001 compared with LPS or LPS/IgG; *P*<0.05 compared LPS/SB with LPS/CD45RB Aβ/SB). For A–E, one-way ANOVA followed by *post hoc* Bonferroni testing was utilized. Note: SB = SB203580, PD = PD98059, Ab = antibody, pp = phosphorylatioin.

It has been reported that the MAPK pathway is central to the biological activities of LPS [28]. This was evidenced by a rapid and transient increase in phosphorylation of both p38 and p44/42 in LPS-stimulated microglial cells. We treated microglial cells with SB203580 (SB, 5 µM; an inhibitor of p38 MAPK) or PD98059 (PD, 5 µM; a selective inhibitor of p44/42) for 1 h prior to treatment with LPS (100 ng/mL) for 30 minutes, and found both inhibitors markedly suppressed the activation of LPS-induced p38 or p44/42 MAPKs (Fig. 2A, B). To investigate whether p38 MAPK and/or p44/42 MAPK are involved in microglial phagocytosis of Aβ_1–42_ peptide, microglial cells were further pre-treated with either of these inhibitors for 1 h, then co-treated with “aged” FITC-tagged Aβ_1–42_ (500 nM) in complete medium for 2 h in the absence (control) or presence of LPS (100 ng/mL). As shown in Fig. 2C (top and bottom panels), both of SB203580 and PD98059 significantly increased microglial phagocytosis of Aβ_1–42_ peptide, with SB230580 showing a more potent effect, a phenomenon which was reversible by LPS stimulation. Together these data indicate p38 MAPK and/or p44/42 MAPK is involved in negative regulation of microglial phagocytosis of Aβ_1–42_ peptide.

### Cross-linking of CD45RB inhibits LPS induced activation of p38 pathway as well as microglial activation

These observations promoted us to investigate the effect of cross-linking CD45RB on LPS-induced p38 MAPK activity in microglial cells. Therefore to determine the signaling mechanism of CD45RB, p38 MAPK phosphorylation was evaluated by Western blot analyses. As shown in Fig. 2D, treatment with LPS (10 ng/mL) for 30 minutes induced significant p38 MAPK phosphorylation. This effect was markedly suppressed by CD45RB antibody (2.5 µg/mL), suggesting that cross-linking CD45RB may attenuate activation of p38 MAPK induced by LPS. We have previously shown that cross-linking of CD45 using an agonistic CD45 antibody leads to inhibition of microglial activation induced by Aβ/CD40 ligation and LPS, as evidenced by TNF-α release and bystander neuronal cell injury [13], [14]. Together with this previous data, it seems that CD45 signaling down-regulates microglial pro-inflammatory responses to LPS challenges. To further investigate whether CD45RB signaling-associated cytokines could be involved in modulating microglial phagocytosis by inhibiting p38 MAPK activity, we examined the effect of cross-linking of CD45RB on the production of TNF-α and IL-6 by measuring their concentrations in culture media supernatant. This effect was mimicked by SB203580. It was shown in Fig. 2E (top and bottom panels) that application of CD45RB antibody (2.5 µg/mL) or SB203580 (5 µM) significantly inhibited LPS-induced TNF-α and IL-6 secretion, and co-treatment with CD45RB antibody and SB203580 results in synergistic inhibition of TNF-α and IL-6 secretion. These data suggest that activation of p38 MAPK is crucial for microglial TNF-α and IL-6 production after challenging LPS, and demonstrate the functionality of CD45RB cross-linking on p38 MAPK activity.

### Inhibition of both p38 and/or p44/42 pathways further enhanced cross-linking CD45RB mediated microglial phagocytosis of Aβ peptide

**Figure 3:**
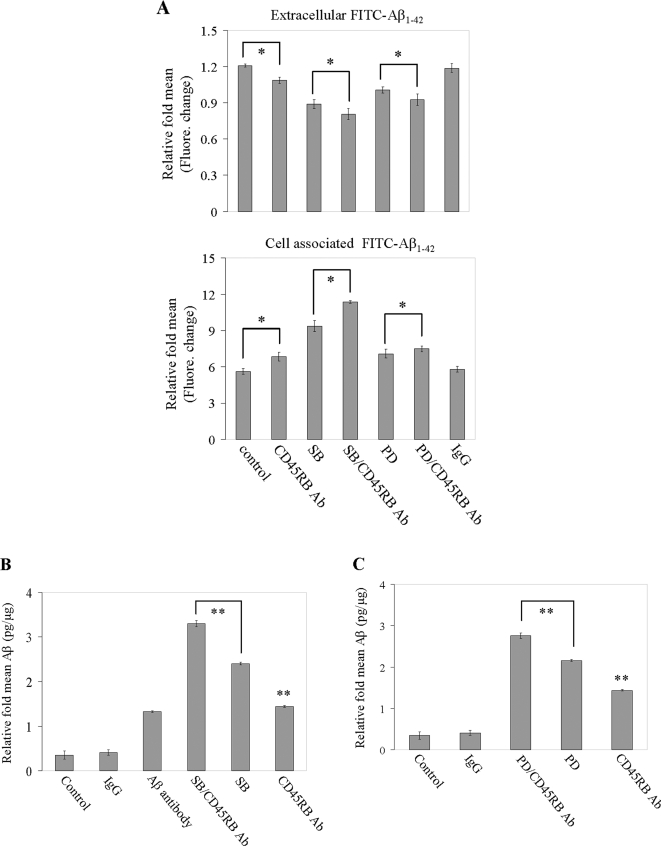
Inhibition of both p38 and/or p44/42 pathways further enhances CD45RB cross-linking mediated microglial phagocytosis of Aβ_1–42_ peptide. Microglial treatment conditions are indicated and are further described in Material and Methods. (A) Cell supernatants and lysates were analyzed for extracellular (top panel) and cell-associated (bottom panel) FITC-Aβ_1–42_ using a fluorometer. Data are represented as the relative fold of mean fluorescence change (mean±SD), calculated as the mean fluorescence for each sample at 37°C divided by mean fluorescence at 4°C (n = 6 for each condition presented). Cell lysates (B, C) were assayed for microglial phagocytosis of Aβ_1–42_ peptide by Aβ-ELISA. Results are reported as picogram per microgram of total protein for cells incubated at 37°C over cells incubated at 4°C. (37°C/4°C; n = 3 for each condition presented). One-way ANOVA followed by *post hoc* Bonferroni testing revealed significant between-group differences (*P<0.05, **P<0.001) and CD45RB Ab compared with isotype-control IgG, *P*<0.001. Note: SB = SB203580, PD = PD98059, Ab = antibody, pp = phosphorylatioin.

Our previous studies have shown either SB203580 or PD98059 inhibitor markedly attenuate CD40 signaling-stimulated activation of p38 or p44/42 MAPK in microglial cells [13], [29]. To determine the role of the p38 and/or p44/42 MAPK cascades in the CD45RB signaling-mediated microglial phagocytosis of Aβ peptide, microglial cells were pre-treated with SB203580 (5 µM) or PD98059 (5 µM) for 1 h, then co-treated with “aged” FITC-tagged Aβ_1–42_ (500 nM) for 2 h in the absence (control) or presence of CD45RB antibody or isotype control IgG. Cell culture supernatants were collected and cell lysates were prepared to measure Aβ by fluorometer (Fig. 3A). In parallel experiments, microglial cells were treated as above. Cell lysates were then prepared and assessed by Aβ ELISA (Fig. 3B for SB203580 and Fig. 3C for PD98059). These data collectively showed that cross-linking CD45RB boosts microglial phagocytosis of Aβ peptide, which was enhanced by inhibition of p38 or p44/42 activities.

### Cross-linking of microglial CD45RB markedly suppresses p38 MAPK activation resulting from phen and Aβ peptide co-treatment

**Figure 4:**
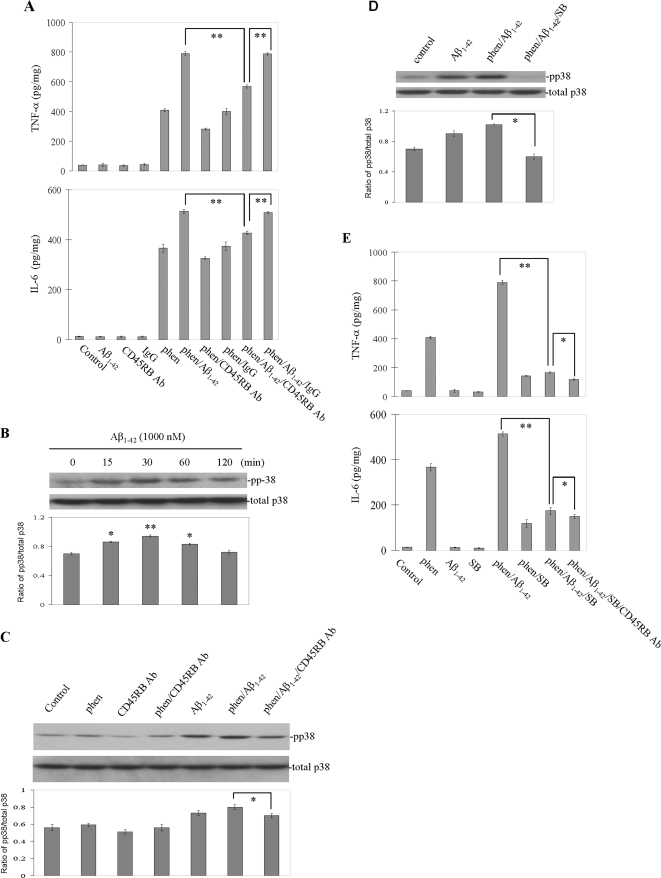
Cross-linking of CD45RB markedly inhibits phen and Aβ peptide-induced microglial activation. (A) Microglial cells were treated as indicated for 16 h. Microglial activation was determined by TNF-α and IL-6 production (mean±1 SD, picograms per milligram of total protein) in cultured media (top and bottom panels). Data are representative of three independent experiments (**P<0.001). (B–D) Cell lysates were analyzed by Western immunoblotting using specific antibodies that recognize phosphorylated p38 MAPK at the indicated time points. Phosphorylation of p38 MAPK is indicated in (B) and inhibition of this effect by CD45RB Ab is indicated in Fig C or by SB in Fig. D. Histograms below the immunoblots represent the mean band density ratio±1 SD (p38/total p38 MAPK; n = 3 for each condition presented). (E) Microglial cells were pre-treated with SB230580 or CD45RB antibody for 1 h, then co-treated with phen and/or aged Aβ_1–42_ for 16 h. Microglial activation was determined by TNF-α and IL-6 production (mean±1 SD) in cultured media (top and bottom panels; n = 3 for each condition presented). One-way ANOVA followed by *post hoc* Bonferroni testing revealed significant differences (*P<0.05, ** *P*<0.001). Note: SB = SB203580, Ab = antibody, pp = phosphorylation).

It has been reported that a tyrosine phosphorylation cascade plays an important role in Aβ-induced microglial activation [30], [31]. Our previous studies demonstrated phen (a specific tyrosine phosphatase inhibitor) synergistically enhanced Aβ-stimulated microglial activation [27]. CD45, a protein-tyrosine phosphatase that is constitutively expressed on microglia [25], is markedly increased on microglia from AD frontal cortices [24], [32]. To investigate the role of CD45RB in microglial activation, we treated primary cultured microglial cells with monoclonal CD45RB antibody before stimulation with phen and Aβ peptide. Microglial activation, as evidenced by TNF-α and IL-6 release after co-treatment of microglia with phen and Aβ peptide, was significantly inhibited by cross-linking CD45RB (Fig. 4A, top and bottom panels). Our previous studies have confirmed this result i*n vivo* [11].

Previous studies have shown that activation of p44/42 MAPK is involved in TNF-α production in macrophages, monocytes, and microglia after activation with a variety of stimuli, including LPS and CD40 ligand [13], [33], [34]. Further our previous studies demonstrated fresh Aβ peptides activate p44/42 MAPK in microglial cells only when CD45 receptor is inactivated with tyrosine phosphatase inhibitors [27]. These data led us to further investigate whether the observed effect of CD45 cross-linking on opposing microglial activation might be mediated *via* activation of the p38 MAPK modulation. First, we analyzed p38 MAPK phosphorylation status. As shown in Fig. 4B, the p38 MAPK pathway was rapid and transiently activated by fresh Aβ. Second, we analyzed activated p38 MAPK status in microglial cell lysates after co-treatment with phen, Aβ, CD45RB antibody or SB203580. Results showed p38 MAPK phosphorylation was induced within 30 minutes after co-treatment with phen and Aβ_1–42_ peptide, which was abrogated by cross-linking of CD45RB or SB203580, respectively (Fig. 4C, D). Furthermore, to determine whether activation of p38 MAPK was responsible for TNF-α and IL-6 production after co-treatment of microglia with phen and Aβ peptide, we treated microglial cells with SB203580 or PD 98059 and CD45RB antibody before stimulation with phen and Aβ peptide. Production of TNF-α and IL-6 were markedly decreased compared with appropriate controls within 16 h after treatment as above (Fig. 4E, top and bottom panels). These data suggest activation of p38 MAPK is crucial for microglial TNF-α and IL-6 production following co-treatment with phen and Aβ peptide, and also suggest cross-linking of CD45RB synergizes with SB203580 to inhibit phen and Aβ-induced activation. In addition, we also observed that cross-linking of CD45RB has the same synergistic role with PD98059 in inhibiting phen and Aβ peptide-induced microglial activation (data not shown).

### Cross-linking of CD45RB inhibits co-localization of microglial MHC class II and Aβ peptide

**Figure 5:**
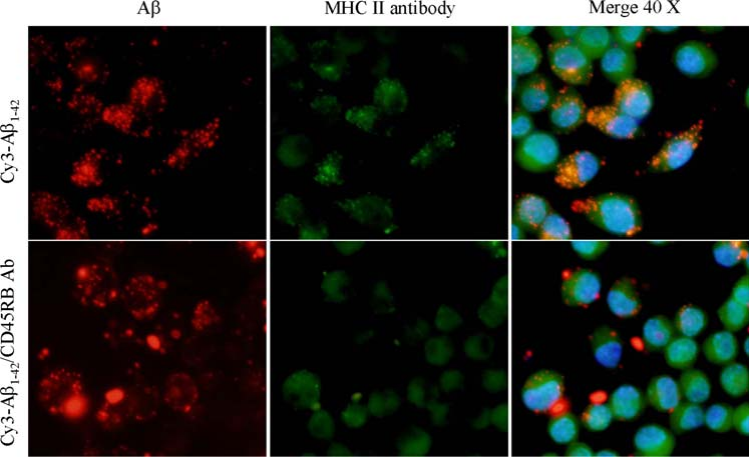
Cross-linking of CD45RB inhibits microglia MHC class II-Aβ co-localization. In order to examine microglia MHC II-Aβ peptide complex formation on the cell surface, microglia were treated with “aged” Cy3-Aβ_1–42_ peptide (300 nM) in the presence or absence of agonist CD45RB antibody for 48 h followed by staining with FITC-anti-mouse MHC class II antibody and Fluorescence microscopy. Note: Red indicates Aβ-positive; green indicates MHC class II-positive; yellow indicates the co-localization of MHC class II and Aβ, Ab indicates antibody. Blue indicates DAPI nuclear stain of the same fields. Original magnification = 40× for top and bottom panels.

MHC class II plays an important role in loading and transporting extracellular pathogens and toxin to the APC surface, where they are recognized by CD^+^ T cells [35]. This cell surface protein is particularly interesting, as microglial cells express it in the frontal cortex and hippocampus of normally aging individuals and levels of expression are markedly increased in these brain regions in AD cases [36], [37]. The impairment of MHC II function results in a significant reduction of microglia-associated CNS inflammation [37], [38], suggesting the elevated level of MHC class II expression is strongly associated with a microglial immune response. Recently, we have shown that CD40 ligation increases MHC II-Aβ peptide complexes as examined by fluorescence microscopy [5]. In addition, we previously showed cross-linking of CD45 significantly opposes CD40-mediated microglial activation [14]. To examine whether cross-linking of CD45RB could inhibit formation of immunogenic MHC II-Aβ peptide complexes on the cell surface, we treated microglia with CD45RB antibody (2.5 µg/mL) in the presence or absence of “aged” Cy3-Aβ_1–42_ peptide (300 nM) for 48 h, followed by immunofluorescence staining with FITC-conjugated anti-mouse MHC class II antibody. Result show that cross-linking of CD45RB inhibits MHC class II-Aβ co-localization as detected by fluorescence microscopy (Fig. 5).

## Discussion

Microglial activation has the potential to be pathogenic in an array of neurodegenerative diseases including multiple sclerosis [39], HIV associated dementia [40], and AD [41], [42]. In the case of AD however, these cells may be therapeutic in clearing brain deposits of aggregated Aβ peptide if induction of a phagocytic state can be achieved [5]. However, current attempts at reducing neuroinflammation mediated *via* microglial activation have only been partially efficacious, possibly because of the fact that such strategies are general inhibitors of inflammation rather specific modulators of the inflammatory versus phagocytic phenotypes of microglia. For example postmortem brain tissue from AD patients who underwent NSAID treatment and control individuals who did not use NSAIDs [43] revealed no significant differences in the mean numbers of senile plaques, senile plaque subtypes (diffuse or neuritic), or neurofibrillary pathology between cases and controls. However the numbers of activated microglia were significantly decreased in NSAID-treated AD patients compared with controls. These data suggest NSAIDs may be therapeutic for AD partially due to their opposition of microglial inflammatory activation. Thus pharmacotherapeutics specifically aimed at blocking microglial inflammatory activation, while promoting microglial Aβ phagocytosis, may hold significant potential for the treatment of Aβ-associated neuropathology in AD. Accumulating evidence has revealed that microglial “activation” is not simply one phenotypic manifestation. The modern view of activated microglia classifies them into two main categories: a phagocytic phenotype (innate activation) or an antigen presenting phenotype (adaptive activation), as governed by their stimulatory environment. Additionally, when challenged with certain pathogen-associated molecular patterns (PAMPs), particularly CpG-DNA, murine microglia seem to activate a “mixed” response characterized by enhanced phagocytosis and pro-inflammatory cytokine production as well as adaptive activation of T cells [44]. Various models of neurodegenerative demonstrate these states of microglial activation whose effects are detectable in human post-mortem brain samples. For example, experimental autoimmune encephalitis model seems to largely support an adaptive, antigen presenting activation of encephalitogenic T cells in the presence of the CD40-CD40 ligand interaction [45]–[47]. Likewise in HIV associated dementia models demonstrate a strong Th1 profile in the CNS [48]. In the context of Aβ challenge in the PSAPP mouse model of AD, CD40 ligation is able to shift activated microglia from innate to adaptive activation. Further, it seems that the cytokine milieu that microglia are exposed to biases these cells to innate activation (i.e., anti-inflammatory Th2-associated cytokines such as IL-4, IL-10, and perhaps TGF-β1) or an adaptive form of activation (i.e., pro-inflammatory Th1-associated cytokines such as IFN-γ, IL-6, and TNF-α) (for review see [49]). Not all forms of microglial activation are deleterious, as activated microglia may serve a protective role as was shown in Aβ_1–42_-immunized mouse models of AD [26], [50], [51]. It seems that enhanced microglial phagocytosis of β-amyloid plaques is at least partly responsible for the therapeutic benefit in these animals, so perhaps stimulation of innate microglial activation contributes to these reported benefits.

In this study, we focused on identifying the signaling mechanisms involved in CD45RB agonism of microglial Aβ phagocytic phenotype. Our rationale for such investigation is based on our previous works in which we have shown the microglial antigen presenting cell (APC)-like phenotype is characterized by pro-inflammatory cytokine secretion, deficient amyloid clearance, and high levels of MHC II expression [5], [11]. This is crucial because when the APC-like phenotype is inhibited, by CD40 neutralizing antibody or pharmacotherapeutics such as statins, microglia reduce their expression of pro-inflammatory neurotoxic cytokines and efficiently phagocytize Aβ from the brain, resulting in accompanying cognitive improvement in mouse models of the disease [11], [12], [14], [52]. Our data show that cross linking of CD45RB enhances microglial phagocytosis of Aβ_1–42_ peptide while suppressing potentially neurotoxic microglia mediated inflammation. This is exemplified by the fact that co-treatment with the PTP inhibitor phen and Aβ peptides resulted in microglial activation as evidenced by increased TNF-α and IL-6 secretion.

p38 is a mitogen-activated protein kinase (MAPK) involved in apoptosis, inflammation and responses to environmental stress [53]. We previously showed that p44/42 inhibition by CD45 agonism is possible [11], and that activation of either p38 or p42/44 pathways leads to a pro-inflammatory microglial phenotype with poor phagocytic ability [5]. Here, we further show that the p44/42 and p38 pathways confer a significant reduction in microglial Aβ phagocytosis and that pre-treatment with selective inhibitors of either p44/42 or p38 significantly upregulates microglial clearance of Aβ peptide (Fig. 2C). We suggest antagonism of p38 by CD45RB stimulation leads to significant reductions in phospho-p38 as shown in figure 2D. However this antagonism of p38 by CD45RB stimulation is likely several folds lower in magnitude than that conferred by the specific p38 inhibitor, SB. Thus the antagonistic effects of CD45RB stimulation on p38 may be undetectable secondary to the very strong p38 antagonism already provided by SB. This may underlie the lack of an additive or synergistic suppression of IL-6 and TNF-α response between the LPS/SB versus LPS/CD45RB Ab/SB groups. Nevertheless, these finding supports a strong positive correlation between inhibition of proinflammatory microglial activation and Aβ phagocytosis as is evident in the significant increase in cell associated Aβ in the SB condition compared to control (Fig. 2C). Furthermore, it was also revealed that p38 MAPK activation is more heavily involved in inhibition of microglial phagocytosis of Aβ_1–42_ peptide than p44/42 (Fig. 2C), which is in agreement with previous studies indicating the central role of p38 in response to inflammatory CNS stimuli [13], [33], [34], [53].

Our previous findings indicated that CD45 inhibits microglial TNF-α release induced by Aβ/CD40 ligation and LPS, [12], [13], [27] as well the reduced microglial phagocytosis under CD45RB suppression. Here, we further show the relative involvement of p38 and p44/42 signaling in CD45RB signal transduction. Specifically, cross-linking of CD45RB in microglia was partially mediated by inhibition of both p38 and p44/42 MAPK activities and hence inversely associated with Aβ phagocytosis. The inhibition of these MAPKs and enhanced Aβ phagocytosis was also supported by an overall shift in microglial phenotype from a pro-inflammatory APC-like state, to an Aβ-clearing phagocytic state as was evident by reduction of MHC class II-Aβ co-localization following the CD45RB cross-linking. MHC class II plays an important role in transport extracellular pathogens and toxin to the APC surface, where they trigger a potentially neurotoxic inflammatory reaction by CD+ T cells. Microglia express it in the frontal cortex and hippocampus of normally aging individuals. Levels of expression are markedly increased in these brain regions in AD cases [36], [37]. Impairment of MHC class II function has previously been shown to induce significant reduction of microglia-associated CNS inflammation (including MS and AD) [37], [38]. An elevated level of MHC class II expression is strongly associated with a pro-inflammatory, non-phagocytic microglia immune response. For example we have shown CD40 ligation results increased MHC class II loaded Aβ peptide complexes associated with reduced amyloid clearance and a neurotoxic elevation of CNS pro-inflammatory cytokines [5].

In summary the AD brain is plagued by a complex inflammatory process, involving neurons and astrocytes, in addition to microglia. It has been suggested the interaction of activated astrocytes and microglia together with the neurons stressed by Aβ peptides sensitize microglia to extracellular stimuli, which then activate MAPK pathways and neurotoxic cytokine release. Indeed transgenic mice overexpressing human amyloid precursor protein (APP) develop early AD-like changes, including diffuse, extracellular Aβ deposits in the brain, and specific spatial learning and pneumonic deficits [54]–[57]. In addition, these animals demonstrate approximately threefold elevation in the number of activated p38 MAPK-positive cells in the brain [58]. More importantly this activity is very specific to microglia with no colocalization with neuronal or astrocytic markers. Indeed many *in vitro* studies have shown fibrillar Aβ peptides induce rapid activation of both p38 and p44/42 MAPKs, resulting in increased TNF-α and NO release [30], [31], [59], [60], while we have shown fresh Aβ peptides activate p44/42 MAPK in microglia only when CD45 receptor is inactivated by PTP [14].

Taken together these data raise the possibility that stimulation of the CD45RB pathway negatively controls microglial activation induced by various proinflammatory stimuli through suppression of both p38 and p42/44 MAPK intracellular signaling systems. Conversely we found CD45RB pathway stimulation positively regulates amyloid clearance suggesting that pharmacotherapeutics targeting stimulation of this receptor may be beneficial in suppressing microglial inflammatory activation and enhancing Aβ clearance; biological correlates which hold strong potential to ameliorate the symptoms of AD.

## Materials and Methods

### Materials

Mouse anti-human Aβ monoclonal antibody (BAM-10) was purchased from Sigma (St. Louis, MO). Aβ_1–42_ and FITC-conjugated Aβ_1–42_ were obtained from Biosource International (Camarillo, CA). Orange fluorescing cyanine dye, Cy3, was purchased from Amersham (Piscataway, NJ) for conjugation with Aβ_1–42_ peptide. Monoclonal (purified rat anti-mouse CD45, including anti-CD45RB, anti-CD45RC, anti-CD45RA, and purified rat IgG2b control antibodies) and purified FITC-anti-mouse MHC class II antibodies were obtained from PharMingen (San Diego, CA). DuoSet™ mouse TNF-α ELISA kit was obtained from R&D systems (Minneapolis, MN). Mouse IL-6 ELISA kit was obtained from eBioscience (San Diego, CA). Antibodies for phospho-p44/42 (pp44/42, Thr202/Tyr204) MAPK, phospho-p38 (pp38, Thr180/Tyr182) MAPK, total p44/42 MAPK, and total p38 MAPK were obtained from Cell Signaling Technology (Beverly, MA) as well as cell lysis buffer, and SDS blue loading buffers. PD98059 (a specific MEK1/2 inhibitor), SB203580 (a specific p38 MAPK inhibitor), and bisperoxo (1, 10-phenanthroline) oxovanadate (phen) were obtained from Calbiochem (La Jolla, CA).

Each of these was dissolved in DMSO before adding to complete cell medium. DMSO alone was used as a solvent control, which did not differ from the untreated controls presented. Bacterial lipopolysaccharide (LPS) was obtained from Sigma (St. Louis, MO) and dissolved in complete cell culture medium. Anti-mouse and anti-rabbit HRP-conjugated IgG secondary antibodies and Western blotting luminol reagent were obtained from Pierce (Rockford, IL). Immun-Blot polyvinylidene difluoride (PVDF) membranes were purchased from Bio-Rad systems (Minneapolis, MN). Anti-Aβ_1–17_ monoclonal antibody (6E10) and biotinylated anti-Aβ_17–24_ monoclonal antibody (4G8) were obtained from Signet Laboratories (Dedham, MA).

### Murine primary cell culture

Breeding pairs of BALB/c mice (Jackson Laboratory, Bar Harbor, ME) were housed in the animal facility at the University of South Florida Health Science Center. Murine primary culture microglia were isolated from mouse cerebral cortices and grown in complete RPMI 1640 medium according to previously described methods [27]. Briefly, cerebral cortices from newborn mice (1–2 days old) were isolated under sterile conditions and kept at 4°C prior to mechanical dissociation. Cells were grown in RPMI 1640 medium supplemented with 5% heat-inactivated FCS, 2 mM glutamine, 100 U/mL penicillin, 100 µg/mL streptomycin, and 50 µM 2-mercaptoethanol. Primary cultures were kept for 14 days so that only glial cells remained. Microglial cells were isolated by shaking flasks at 200 rpm in a Lab-Line™ Incubator-Shaker. More than 98% of these glial cells stained positive for Mac-1 (Boehringer Mannheim, Indianapolis, IN).

### Microglial phagocytosis assays

Primary mouse microglia were seeded at 1×10^5^ cells/well (n = 6 for each condition) in 24-well tissue culture plates containing 0.5 mL of complete RPMI 1640 medium. These cells were treated for 2 h with “aged” Aβ_1–42_ conjugated with FITC (Biosource International; 500 nM pre-aggregated for 24 h at 37°C in complete medium as described by [61]). In the presence of FITC-Aβ_1–42_, microglial cells were then co-treated with agonist CD45, CD45RB, CD45RC, and CD45RA antibodies, or isotype control IgG (2.5 µg/mL). Some of these cells were treated with PD98059 (5 µM) or SB203580 (5 µM) for 1 h prior to LPS (100 ng/mL), or CD45RB antibody (2.5 µg/mL), isotype control IgG (2.5 µg/mL) or BAM-10 (2.5 µg/mL), in the presence of FITC-Aβ_1–42_ for 2 h. Microglial cells were then rinsed three times in Aβ-free complete medium, and the media were exchanged with fresh Aβ-free complete medium for 10 minutes to allow for removal of non-incorporated Aβ and promote concentration of Aβ into phagosomes. Extracellular and cell-associated FITC-Aβ were quantified using an MSF (SpectraMax®, Molecular Devices) with an emission wavelength of 538 nm and an excitation wavelength of 485 nm. A standard curve from 0 to 600 nM of FITC-Aβ was run for each plate. Total cellular proteins were quantified using the Micro BCA Protein Assay (Pierce, Rockford, IL). The mean fluorescence values for each sample at 37°C and 4°C at the 2 h point were determined by fluorometric analysis. Relative fold change values were calculated as: mean fluorescence value for each sample at 37°C/mean fluorescence value for each sample at 4°C. In this manner, both extracellular and cell-associated FITC-Aβ were quantified. Considering nonspecific adherence of Aβ to the plastic surface of culture plates, an additional control without cells was carried out through all of experiments above. An incubation time of less than 4 h did not change the amount of Aβ peptide detected in the supernatant, which is consistent with a previous report [62]. In order to determine the extent to which cell death might have influenced the phagocytic activity in the various treatment groups, we performed the LDH assay on the relevant supernatant. Data showed that there was no significant cell death occurring over the 3 h time frame in any of the treatment groups (data not shown, *P*<0.05).

### Fluorescence microscope examination

“Aged” FITC-Aβ_1–42_ was prepared according to methods described above. Microglial cells were cultured at 1×10^5^ cells/well in 24-well tissue culture plates with glass inserts. In the presence of FITC-Aβ_1–42_ (1 µM), microglial cells were co-treated with agonist CD45RB antibody (2.5 µg/mL) or isotype control IgG (2.5 µg/mL) at 37°C for 2 h. Additionally, in parallel 24-well tissue culture plates, microglial cells were incubated at 4°C with the same treatment as above. Following treatment, these cells were washed five times with ice-cold phosphate buffered saline (PBS) to remove extracellular Aβ and fixed for 10 minutes at 4°C in 4% (w/v) paraformaldehyde (PFA) diluted in PBS, followed by staining with 4′,6-Diamidino-2-Phenylindole (DAPI) at 4°C for 15 minutes. Finally sections were mounted with fluorescence mounting media containing Slow Fade antifading reagent (molecular Probes, Eugene, OR), and then viewed under an Olympus IX71/IX51 microscope equipped with a digital camera system (40×). Cy3-Aβ_1–42_ was prepared according to previous methods [61]. Primary microglial cells (seeded in 24-well tissue culture plates at 5×10^4^ cells/well) were treated with Cy3-Aβ_1–42_ peptide (300 nM) in the presence or absence of agonist CD45RB antibody at 37°C for 48 h. Following treatment, these cells were washed, fixed, and permeabilized in 0.2% triton X-100, 5% horse serum for 1 h. This was followed by staining with FITC-anti-mouse MHC class II antibody (2 µg/mL incubated overnight at 4°C). Slides were analyzed using the same microscope equipped as above.

### Fluorescence confocal microscopy

In parallel with the Fluorescence microscope examination, slides were viewed with a Leica DMI6000 inverted microscope, TCS SP5 confocal scanner, and 63×/1.4NA Plan Apochromat oil immersion objectives (Leica Microsystems, Germany). Excitation wavelengths of 488 nm (for FITC) and 405 nm (for DAPI) were used to generate fluorescence emission in green (for Aβ_1–42_) and blue (for nuclei), respectively. Images were captured with photomultiplier detectors at 3× zoom and were prepared with the LAS AF software version 1.6.0 build 1016 (Leica Microsystems, Germany). DIC image were also captured using the 488 laser line.

### TNF-α, IL-6, and Aβ ELISA

#### TNF-α and IL-6 ELISA

Primary cultured microglial cells were plated in 24-well tissue culture plates (Costar, Cambridge, MA) at 5×10^4^ cells/well for 24 h. They were then stimulated for 16 h with phen (5 µM), Aβ peptides (1 µM), phen+Aβ peptides, or LPS (10 ng/mL) in the presence or absence of pretreatment (1 h) with agonist CD45RB antibody (2.5 µg/mL), PD98059 (5 µM), SB203580 (5 µM), or appropriate controls. Cell-free supernatants were collected and assayed by TNF-α (Minneapolis, MN), and IL-6 (San Diego, CA) ELISA kits in strict accordance with the manufacturer's instruction. The BCA Protein Assay (Pierce, Rockford, IL) was performed to measure total cellular protein from each of the cell groups under consideration just before quantification of cytokine release.

#### Aβ ELISA

Primary mouse microglial cells were seeded at 1×10^5^ cells/well (n = 6 for each condition) in 24-well tissue culture plates containing 0.5 mL of complete RPMI 1640 medium. These cells were incubated at 37°C and 4°C with the same treatment conditions as microglial phagocytosis assays. Cell lysates were prepared and quantization of microglial phagocytosis of total Aβ peptides was performed according to published methods [63]. Briefly, 6E10 (capture antibody) was coated at 2 µg/mL in PBS in 96-well immunoassay plates overnight at 4°C. The plates were then washed with 0.05% Tween-20 in PBS five times and blocked with blocking buffer (PBS with 1% bovine serum albumin, 5% horse serum) for 2 h at room temperature. Conditioned medium or Aβ standards were added to the plates and incubated overnight at 4°C. Following three washes, biotinylated antibody 4G8 (0.5 µg/mL in PBS with 1% bovine serum albumin) was added to the plates and incubated for 2 h at room temperature. After five washes, streptavidin-horseradish peroxidase (1∶200 dilutions in PBS with 1% bovine serum albumin) was added to the 96-well plates for 30 minutes at room temperature. Tetramethylbenzidine substrate was then added to the plates and incubated for 15 minutes at room temperature. Then 50 µL of stop solution (2 N H_2_SO_4_) was added to each well of the plates. The optical density of each well was immediately determined by a microplate reader at 450 nm. In addition, Aβ_1–42_ was quantified in these samples using the Aβ_1–42_ ELISA kits (IBL-America, Minneapolis, MN) in accordance with the manufacturer's instructions.

### Western immunoblotting

Murine primary culture microglia were plated in six-well tissue culture plates at a density of 8×10^5^ cells/well. These cells were incubated for 30 minutes with or without LPS (10 ng/mL), Aβ_1–42_ (1 µM), phen (5 µM)+Aβ_1–42_ (1 µM), in the presence or absence of pretreatment (1 h) with CD45RB antibody, isotype control IgG (2.5 µg/mL), PD98059 (5 µM), SB203580 (5 µM), or appropriate controls. After treatment, microglial cells were washed in ice-cold PBS three times. Next the cells were lysed with 1× SDS sample buffer [62.5 mM Tris-HCI (pH6.8), 2% SDS, 10% glycerol, 50 mM dithiothreitol], sonicated for 15 seconds, and then heated to 100°C for 5 minutes. The cell lysates were centrifuged at 12,000 rpm (4°C) for 5 minutes, and the protein concentration of the supernatant was measured by BCA Protein Assay System (Pierce). Western blotting of phosphorylated p38 or p44/42 MAPK was performed according to the manufacturer's instructions using phospho-specific antibodies. Briefly, proteins were electrophoresed on a 10% SDS-PAGE gels and transferred to immunoblotting PVDF membranes (Bio-Rad). The membranes were blocked for 1 h at room temperature in Tris-buffered saline (TBS, Bio-Red), 0.1% Tween-20 with 5% nonfat dry milk, and then were incubated with primary antibodies overnight at 4°C. After incubation with horseradish peroxidase-conjugated secondary antibody, the protein bands were detected with a Super Signal west Femto Maximum Sensitivity Substrate (Pierce) and BIOMAX-MR film (Eastman Kodak Co.). For detection of total p38 and p44/42, the membranes were stripped with Restore Western blot Stripping Buffer (Pierce) followed by incubation with specific antibodies.

### Statistical analysis

All experiments were performed at least three times, and the representative results were shown. Data were analyzed using ANOVA followed by *post hoc* comparisons of means by Bonferroni's. A value of *P*<0.05 was considered statistically significant.
